# Neuromuscular Blockade for Cardiac Arrest Patients Treated With Targeted Temperature Management: A Systematic Review and Meta-Analysis

**DOI:** 10.3389/fphar.2022.780370

**Published:** 2022-05-24

**Authors:** Tong Lin, Yan Yao, Yuan Xu, Hui-Bin Huang

**Affiliations:** ^1^ Department of Reproductive Endocrinology, Hospital of Traditional Chinese Medicine, Zhaoqing, China; ^2^ Department of Critical Care Medicine, School of Clinical Medicine, Beijing Tsinghua Changgung Hospital, Tsinghua University, Beijing, China

**Keywords:** neuromuscular-blocking agents, cardiac arrest, targeted temperature management, neurological outcome, meta-analysis

## Abstract

**Background:** Neuromuscular-blocking agents (NMBA) are often administered to control shivering in comatose cardiac arrest (CA) survivors during targeted temperature management (TTM) management. Thus, we performed a systematic review and meta-analysis to investigate the effectiveness and safety of NMBA in such a patient population.

**Methods:** We searched for relevant studies in PubMed, Embase, and the Cochrane Library until 15 Jul 2021. Studies were included if they reported data on any of the predefined outcomes in adult comatose CA survivors managed with any NMBA regimens. The primary outcomes were mortality and neurological outcome. Results were expressed as odds ratio (OR) or mean difference (MD) with an accompanying 95% confidence interval (CI). Heterogeneity, sensitivity analysis, and publication bias were also investigated to test the robustness of the primary outcome.

**Data Synthesis:** We included 12 studies (3 randomized controlled trials and nine observational studies) enrolling 11,317 patients. These studies used NMBA in three strategies: prophylactic NMBA, bolus NMBA if demanded, or managed without NMBA. Pooled analysis showed that CA survivors with prophylactic NMBA significantly improved both outcomes of mortality (OR 0.74; 95% CI 0.64–0.86; *I* ^2^ = 41%; *p* < 0.0001) and neurological outcome (OR 0.53; 95% CI 0.37–0.78; *I* ^2^ = 59%; *p* = 0.001) than those managed without NMBA. These results were confirmed by the sensitivity analyses and subgroup analyses. Only a few studies compared CA survivors receiving continuous versus bolus NMBA if demanded strategies and the pooled results showed no benefit in the primary outcomes between the two groups.

**Conclusion:** Our results showed that using prophylactic NMBA strategy compared to the absence of NMBA was associated with improved mortality and neurologic outcome in CA patients undergoing TTM. However, more high-quality randomized controlled trials are needed to confirm our results.

## Introduction

Targeted temperature management (TTM) has been demonstrated to improve the neurological prognosis of survivors after resuscitation for cardiac arrest (CA) and is recommended by clinical guidelines (Callaway et al., 2015). However, shivering, one of the most common complications during TTM, can counteract the beneficial effects of TTM by generating heat, increasing metabolic rate and oxygen consumption, preventing the rapid achievement of target temperatures, and causing secondary brain injury (Seder et al., 2011). Therefore, shivering should be avoided or controlled as early as possible during TTM.

Neuromuscular-blocking agents (NMBA) can effectively reduce the occurrence of shivering and are widely used in clinical practice (Greenberg and Vender, 2013). Theoretically, NMBA can also improve chest wall compliance and eliminate patient-ventilator asynchrony; reduce cerebral metabolic demand, shorten the time to target temperature, and prevent the increase in intracranial pressure caused by airway stimulation (Greenberg and Vender, 2013; deBacker et al., 2017). However, NMBA is not without risks. Several studies have reported that NMBA treatment is associated with increased risks of nosocomial pneumonia (Lascarrou et al., 2014) and critical illness polyneuromyopathy (Price et al., 2012). In addition, NMBA treatment may mask epileptic activity and limit neurological evaluation (Al-Dorzi et al., 2012). The 2015 American Heart Association (AHA) recommended that NMBA should be minimized or avoided during post-CA care (Callaway et al., 2015). Thus, whether NMBA affects the outcome of survivors after CA remains unclear.

Recently, several studies on this topic have been published (Stöckl et al., 2017; Lee et al., 2018; Moskowitz et al., 2020; Hifumi et al., 2021; Takiguchi et al., 2021), and some of these have a modest sample size with inconsistent results. This may be related to the different strategies, timing, and research design of NMBA applications. Therefore, we sought to conduct a systematic review and meta-analysis by pooling existing studies to investigate the efficacy and safety of NMBA strategy in CA survivors during TTM.

## Methods

We conducted this systemic and meta-analysis following the Preferred Reporting Items for Systematic Reviews and Meta-Analyses (PRISMA) statement (Shamseer et al., 2015). (Supplementary Additional File S1). The protocol for this systematic review and meta-analysis was registered on the International Platform of Registered Systematic Review and Meta-analysis Protocols database (INPLASY202070045) and is available in full on inplasy.com (https://doi.org/10.37766/inplasy2020.7.0045).

### Search Strategy and Selection Criteria

We searched studies in PubMed, Embase, and Cochrane Library from inception through 25 Jul 2021, to identify potentially relevant studies. Language restriction was limited in English and Chinese. We also reviewed reference lists of relative articles. Details of the search strategy are provided in Supplementary Additional File S2.

After screening titles, we evaluated abstracts for relevance and identified them as included, excluded, or requiring further assessment. Studies were considered for inclusion if they focused on CA survivors during TTM and compared different NMBA strategies, including but not limited to prophylactic NMBA (continuous or scheduled), bolus if demanded or managed without NMBA (defined as the use of placebo, saline, or no use; patients are allowed to receive emergent NMBA use to control shivering episodes). We excluded studies enrolling children, pregnant women, or patients with pre-existing dementia or brain injury. Articles published in editorials, narrative reviews without data on predefined outcomes available were also excluded.

### Data Extraction and Quality Assessment

Two reviewers (L-JL and H-BH) independently extracted data from the included studies on the first author, year of publication, country, sample size, study design, disease severity, NMBA and TTM regimens, methodological quality, and all outcomes of interest. L-JL and H-BH also evaluated the quality of included studies using the risk of bias tool recommended by the Cochrane Collaboration in randomized clinical trials (RCTs) (Higgins et al., 2011) and the Newcastle-Ottawa scale for assessing the risk of bias in observational studies (Stang, 2010). Discrepancies were identified and resolved through discussion.

### Predefined Outcomes

We aimed to explore the effectiveness and safety of NMBA strategies during TTM, including 1) with or without NMBA strategy; and 2) NMBA administration methods (i.e., continuous vs. intermittent). The primary outcomes were mortality at the longest follow-up available and the neurological outcome. Secondary outcomes included duration of MV, ICU or hospital stay, lactate clearance, time to targeted temperature, and NMBA associated complications (i.e., pneumonia).

### Statistical Analysis

The results from all relevant studies were combined to estimate the pooled risk ratio (RR) and associated 95% confidence intervals (CIs) for dichotomous outcomes. As to the continuous outcomes, mean differences (MD) and 95% CI were estimated as the effect results. We assessed heterogeneity using the Mantel-Haenszel χ two test and the *I* ^2^ statistic (Higgins et al., 2003). An *I* ^2^ < 50% was considered to indicate insignificant heterogeneity, and a fixed-effect model was used, whereas a random-effect model was used in cases of significant heterogeneity (*I* ^2^ > 50%). Before data analysis, we estimated mean from median and standard deviations (SD) from IQR using the methods described in previous studies (Wan et al., 2014). We conducted subgroup analyses basing NMBA strategies. Sensitivity analyses were performed by excluding trials that potentially biased the results of primary outcomes. We also conducted sensitivity analyses for the primary outcomes by pooling only RCTs or studies focusing on targeted temperature of 32–34°C to investigate the potential affecting factors among the included studies. Publication bias was evaluated by visually inspecting funnel plots. All analyses were performed using Review Manager version 5.3.

## Results

### Study Selection

**FIGURE 1:**
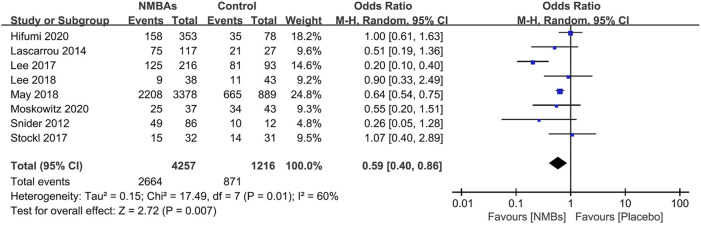
The selection process for studies included in the meta-analysis.

The literature search yielded 881 records through database searching, and 12 studies with 11,317 patients who fulfilled inclusion criteria were eligible for final analysis (Jurado and Gulbis, 2011; Snider et al., 2012; Salciccioli et al., 2013; Curtis et al., 2014; Lascarrou et al., 2014; Lee et al., 2017; Stöckl et al., 2017; Lee et al., 2018; May et al., 2018; Moskowitz et al., 2020; Hifumi et al., 2021; Takiguchi et al., 2021). Additionally, in replying to the letter comment on their study (Salciccioli et al., 2013), Salciccioli et al. provided some related data Salciccioli and Donnino, (2014), which were also included in our meta-analysis. The overview of the study selection process is presented in Figure 1.

### Study Characteristics

The main characteristics of the 12 included studies [3 RCTs (Stöckl et al., 2017; Moskowitz et al., 2020; Lee et al., 2018) and nine observational studies (Lascarrou et al., 2014; Takiguchi et al., 2021; Hifumi et al., 2021; Salciccioli et al., 2013; May et al., 2018; Lee et al., 2017; Jurado and Gulbis, 2011; Curtis et al., 2014; Snider et al., 2012)] are shown in Table 1. The NMBA regimens described in the included studies were presented in Supplementary Additional File S3. Of these studies, Six and five were single-center (Jurado and Gulbis, 2011; Snider et al., 2012; Curtis et al., 2014; Lascarrou et al., 2014; Lee et al., 2017; Stöckl et al., 2017) and multi-center studies (Salciccioli et al., 2013; Lee et al., 2018; May et al., 2018; Moskowitz et al., 2020; Hifumi et al., 2021), respectively, and one used data from an international cardiac arrest registry (May et al., 2018). Nine studies (Snider et al., 2012; Curtis et al., 2014; Lascarrou et al., 2014; Lee et al., 2017; Stöckl et al., 2017; Lee et al., 2018; May et al., 2018; Moskowitz et al., 2020; Hifumi et al., 2021) compared patients receiving prophylactic NMBA with the absence of NMBA regimen, and five (Jurado and Gulbis, 2011; Salciccioli et al., 2013; Lee et al., 2018; May et al., 2018; Takiguchi et al., 2021) evaluated the effects of continuous NMBA with bolus NMBA if demanded. The duration of NMBA used ranged from 24 to 37 h among studies. Most studies assessed neurological outcome based on Cerebral Performance Category score (CPC), with good outcome defined as CPC of one or two and poor outcome as CPC of 3–5. Sedation and anesthetic schemes varied across the included studies and were summarized in the Supplementary Additional File S4. Overall, the quality of the included studies was low to medium (Supplementary Additional File S5).

**TABLE 1:** Characteristics of the studies included in current systemic review and meta-analysis.

Study	Design	Country	TTM,°C	NMB Regimens	OHCA, %	Sample size	Age, year	Male %	Defined good neurological outcome	Follow**-** **up**
Moskowitz et al. (2020)	RCT, MC	United States	32–36	Prophylactic	95	37	66	54	mRS score of 0–3	Hospitalization
				As-needed	93	43	64	67		
Hifumi et al. (2021)	R, MC	Japan	32–34	Prophylactic	100	353	61	80.5	CPC of 1–2	Hospitalization
				No use	100	78	60	91		
Takiguchi et al. (2021)	R, DB	Japan	<35	Prophylactic	91	4,096	59	78	Barthel index score >85	Hospitalization
				As-needed	88	1,488	62	76		
Lee et al. (2018)	RCT, MC	Korea	33 or 36	Prophylactic	100	38	66	29	CPC of 1–2	Hospitalization
				No use	100	43	61	30		
May et al. (2018)	P, MC	United States	32–34	Prophylactic	81	1,462	60	65	CPC of 1–2	6 months
				As-needed	75	1,916	61	70		
				No use	72	889	65	62		
Lee et al. (2017)	R, SC	Korea	32–34	Prophylactic	79	97	57	75	CPC of 1–2	Hospitalization
				As-needed	99	119	60	77		
				No use	70	93	66	46		
Stöckl et al. (2017)	RCT, SC	Austria	33	Prophylactic	100	32	62	26	CPC of 1–2	12 months
				No use	100	31	58	26		
Lascarrou et al. (2014)	R, SC	France	33	Prophylactic	82	117	59	94	CPC of 1–2	3 months
				No use	93	27	66	19		
Curtis et al. (2014)	R, SC	United States	32–34	Prophylactic	NA	19	57	NA	NA	NA
				No use	NA	7	56	NA		
Snider et al. (2012)	R, SC	United States	34	Prophylactic	NA	86	NA	NA	CPC of 1–2	Hospitalization
				No use	NA	12	NA	NA		
Salciccioli et al. (2013)	P, MC	United States	34	Prophylactic	100	18	56	14	mRS score of 0–3	Hospitalization
				As-needed	100	77	NA	Na		
				No use	100	16	NA	NA		
Jurado and Gulbis, (2011)	R, SC	United States	33	Prophylactic	NA	80	58	65	NA	Hospitalization
				As-needed	NA	43	57	35		

CPC, cerebral performance category; DB, data base; MC, multi-centers; mRS, modified Rankin Scale; NA, not available; NMB, neuromuscular blockade; OHCA, out-of-hospital cardiac arrest; P, prospective; R, retrospective; RCT, randomized controlled trials; SC, single-center; TTM, time to target temperature.

### Primary Outcomes

#### With or Without NMBA Regimen

**FIGURE 2:**
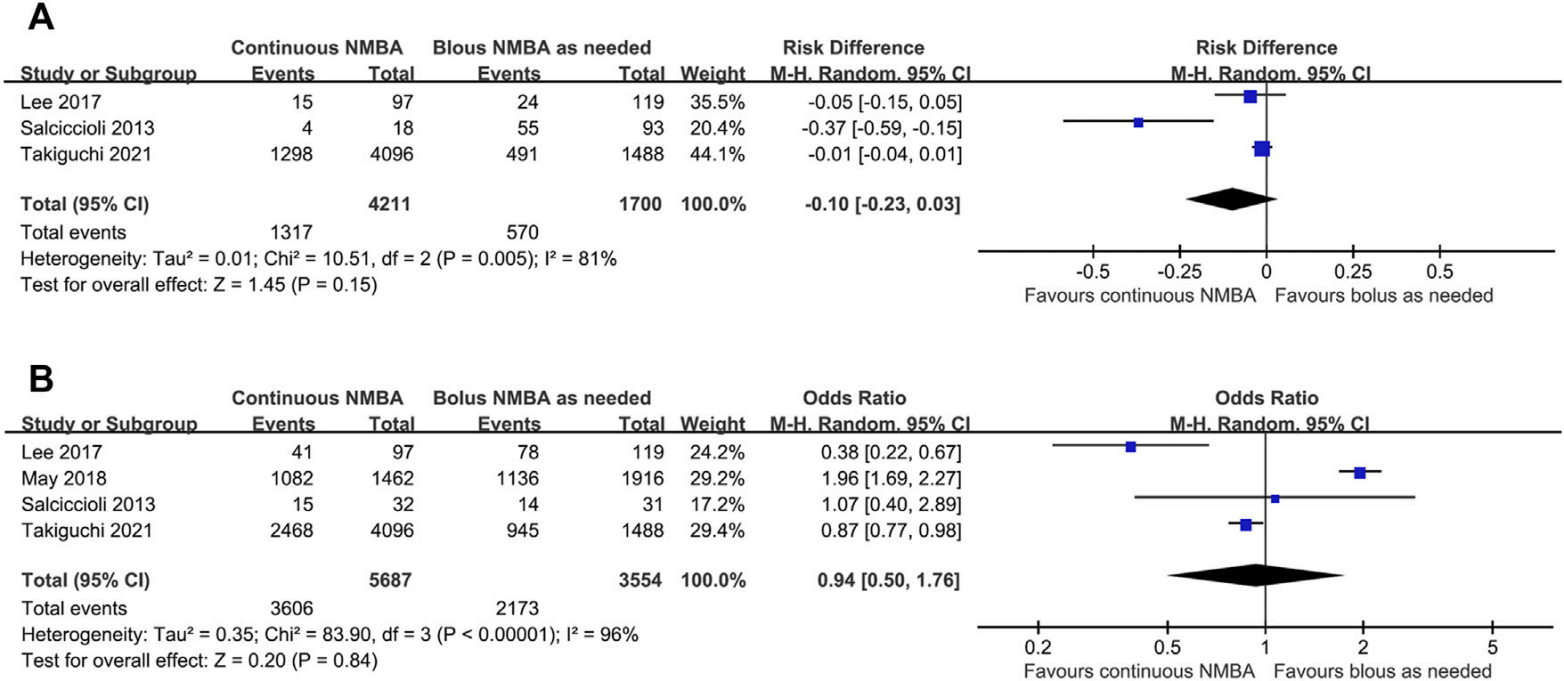
Forest plot of comparing prophylactic neuromuscular-blocking agent to without neuromuscular-blocking agent regimen in outcomes of mortality.

Nine studies with 5,410 patients compare prophylactic NMBA (scheduled or continuous) to without NMBA regimen (Snider et al., 2012; Curtis et al., 2014; Lascarrou et al., 2014; Lee et al., 2017; Stöckl et al., 2017; Lee et al., 2018; May et al., 2018; Moskowitz et al., 2020; Hifumi et al., 2021). Eight of these studies reported outcomes of mortality (Salciccioli et al., 2013; Curtis et al., 2014; Lascarrou et al., 2014; Lee et al., 2017; Stöckl et al., 2017; Lee et al., 2018; Moskowitz et al., 2020; Hifumi et al., 2021), and the aggregated data suggested that the mortality was significantly lower in the prophylactic NMBA (*n* = 1,245; OR 0.74; 95% CI 0.64–0.86; *I* ^2^ = 41%; *p* < 0.0001) when compared to without NMBA regimen (Figure 2). Subgroup analyses confirmed that continuous NMBA, bolus NMBA, or combined with continuous and bolus have significantly lower mortality rates (Table 2, Supplementary Additional File S6).

**TABLE 2:** Subgroup analysis of the mortality and Poor neurological outcomes based on NMB regimens.

Subgroup		Included studies, [Reference]	Sample size	Event in apheresis group	Event in control group	Odd ratio (95% CI)	*P*	*I* ^2^ %
Mortality	Continue vs. no use	(Lascarrou et al., 2014), (Lee et al., 2017), (Stöckl et al., 2017), (Moskowitz et al., 2020), (Lee et al., 2018), (Salciccioli et al., 2013)	592	144/339	118/237	0.53 [0.37, 0.77]	0.0008	50
	Bolus as need/continuous vs. no use	(Hifumi et al., 2021), (Curtis et al., 2014), (Salciccioli et al., 2013)	568	127/467	38/101	0.53 [0.37, 0.77]	0.01	16
	Bolus as need vs. no use	(Lee et al., 2017), (Salciccioli et al., 2013)	305	69/196	45/109	0.49 [0.29, 0.84]	0.01	0
Poor neurological outcome	Continue vs. no use	(Lascarrou et al., 2014), (Stöckl et al., 2017), (Salciccioli et al., 2013), (May et al., 2018), (Lee et al., 2017)	2,978	1228/1,845	847/1,133	0.80 [0.64, 1.00]	0.05	0
	Bolus/continuous vs. no use	May et al. (2018)	529	207/439	45/90	0.83 [0.55, 1.25]	0.49	77
	Bolus as need vs. no use	(Hifumi et al., 2021), (Snider et al., 2012)	2,805	718/1,916	664/889	0.50 [0.47, 0.54]	<0.0001	-

**FIGURE 3:**
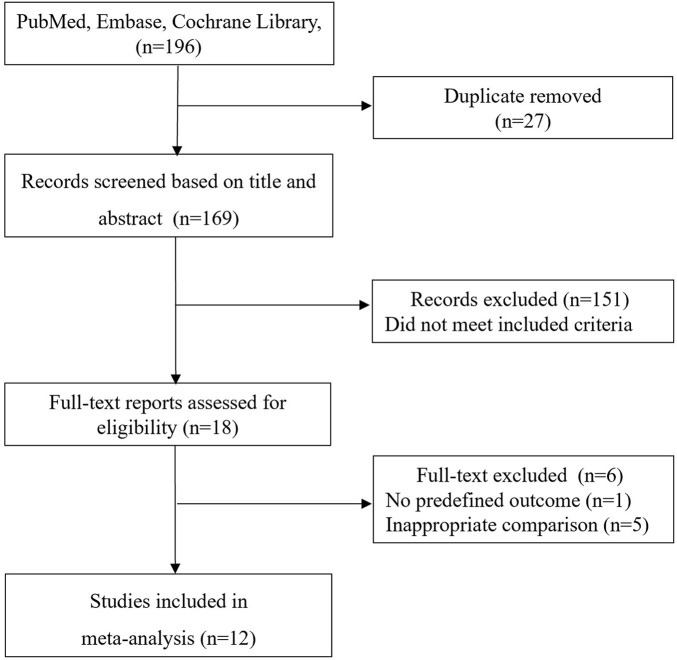
Forest plot of comparing prophylactic neuromuscular-blocking agent to without neuromuscular-blocking agent regimen in poor neurological outcome.

Seven studies focused on the neurological outcomes (Snider et al., 2012; Lascarrou et al., 2014; Lee et al., 2017; Stöckl et al., 2017; Lee et al., 2018; May et al., 2018; Moskowitz et al., 2020; Hifumi et al., 2021). Pooled analysis showed the poor neurological outcome was significantly lower in the prophylactic NMBA group than that of without NMBA (*n* = 5,521; OR 0.53; 95% CI 0.37–0.78; *I* ^2^ = 59%; *p* = 0.001) (Figure 3). The subgroup analyses showed significant reductions in poor neurological outcomes in patients receiving continuous NMBA or bolus NMBA but not combining continuous and bolus NMBA strategies (Table 2, Supplementary Additional File S6).

In the sequential sensitivity analysis, excluding any single test did not significantly change the overall combined OR for the outcome of mortality (*p* < 0.00001–0.03) and neurological outcome (*p* < 0.00001–0.04). When a sensitivity analysis including only RCTs was performed, the results for both outcomes were not significantly in favor of prophylactic NMBA for outcomes of mortality (RR 0.95; 95% CI 0.74–1.21; *p* = 0.68) and neurological outcome (RR 0.91; 95% CI 0.71–1.17; *p* = 0.48), with the heterogeneity disappeared. When a sensitivity analysis including only studies focusing on targeted temperature of 32–34°C were performed, the results for both outcomes were also similar to the results including all studies (mortality: RR 0.71; 95% CI 0.55–0.92; *p* = 0.009, *I* ^2^ = 50% and neurological outcome: RR 0.81; 95% CI 0.70–0.94; *p* = 0.005, *I* ^2^ = 72%), while the heterogeneity existed.

#### Continuous vs. Bolus NMBA

**FIGURE 4:**
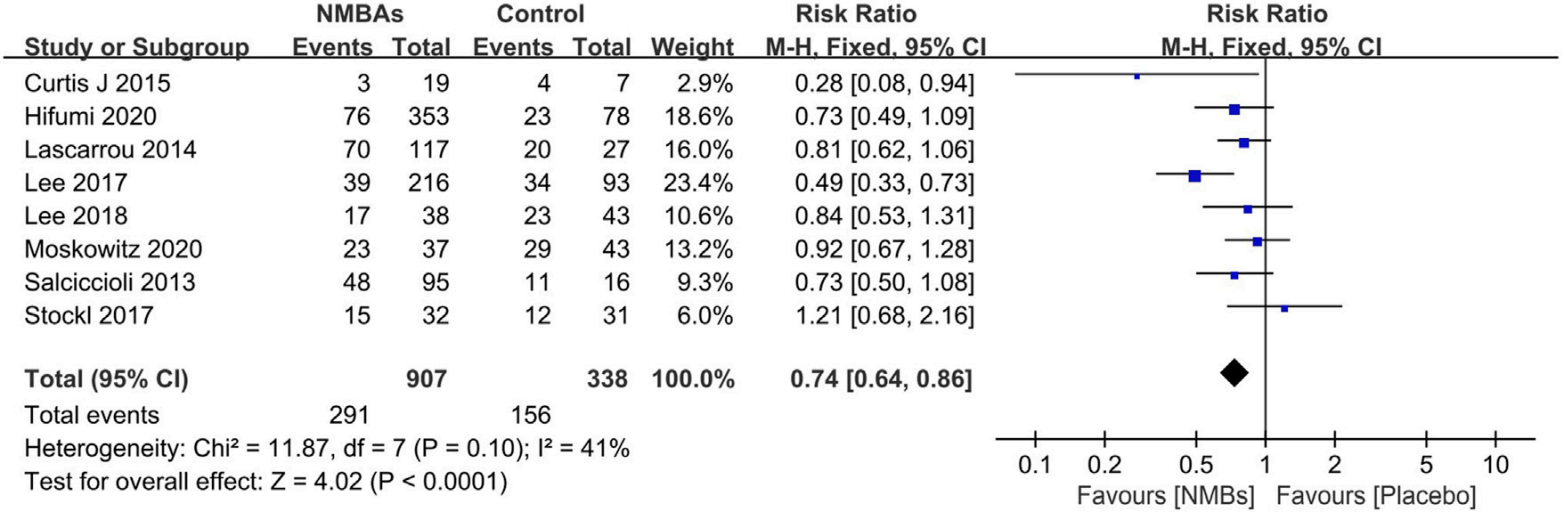
Forest plot of comparing continuous neuromuscular-blocking agent to bolus neuromuscular-blocking agent regimen in outcomes of mortality **(A)** and poor neurological outcome **(B)**.

Five studies examined the efficacy of continuous NMBA compared to bolus NMBA if demanded (Takiguchi et al., 2021; Salciccioli et al., 2013; May et al., 2018; Lee et al., 2017; Jurado and Gulbis, 2011). Pooled data showed no statistically significant difference between the two regimens in the risk of mortality (3 studies; *n* = 5,911; OR, −0.10; 95% CI, −0.23 to 0.03; *I* ^2^ = 81%; *p* = 0.15) (Takiguchi et al., 2021; Salciccioli et al., 2013; Lee et al., 2017) (Figure 4A) or poor neurological outcome (4 studies; *n* = 9,241; OR, 0.94; 95% CI, 0.50–1.76; *I* ^2^ = 96%; *p* = 0.84) (Salciccioli et al., 2013; Lee et al., 2017; May et al., 2018; Takiguchi et al., 2021) (Figure 4B). We did not perform the subgroup analysis for the limited studies for both outcomes. In the sequential sensitivity analysis, the results for both outcomes were confirmed by excluding any single test. When a sensitivity analysis including only studies focusing on targeted temperature of 32–34°C were performed, the results for both outcomes were also similar to the results including all studies (mortality: RR 0.44; 95% CI 0.13–1.41; *p* = 0.009, *I* ^2^ = 66% and neurological outcome: RR 0.59; 95% CI 0.22–1.58; *p* = 0.005, *I* ^2^ = 68%).

### Secondary Outcomes

When comparing the prophylactic NMBA and without NMBA regimen, we found prophylactic NMBA strategy benefited more in CA survivors who received TTM in the outcomes of time to achieve target temperature and length of hospital stay. The duration of MV, serum lactate clearance after 24 h, and pneumonia incidence were similar between groups. Few studies compared continuous and intermittent NMBA regimens and showed continuous NMBA regimens had significantly longer ICU stay and shorter length of MV than intermittent NMBA regimens. (Table 3).

**TABLE 3:** Secondary outcomes of the current systematic review and meta-analysis.

Secondary outcome	Included studies, [Reference]	Sample Size	Odd ratio/Mean difference [95% CI]	*P*	*I* ^2^%	Included studies, [Reference]	Sample Size	Odd ratio/Mean difference [95% CI]	*P*	*I* ^2^%
Prophylactic NMBA vs. without NMBA regimens						Continuous infusion vs. intermittent bolus NMBA regimens				
Length of stay in ICU	9–11,20,18	725	0.80 [−0.87, 2.46]	0.35	76	20,21	339	3.79 [−2.57, 5.01]	<0.0001	0
Length of stay in hospital	11,18	192	3.11 [0.46, 5.76]	0.02	0	8	5584	−3.00 [−6.24, 0.24]	0.07	-
Incidence of pneumonia	5,12	576	0.59 [0.40, 0.86]	0.55	87	8	5584	0.87 [0.73, 1.05]	0.15	-
Duration of MV	5,9,10,11	644	0.15 [−1.15, 1.45]	0.82	0.85	8,20	5800	−2.17 [−4.10, −0.24]	0.03	70%
Change of lactate after 24 h	9,10,18,19	418	0.31 [−0.33, 0.96]	0.34	0	20	216	-	>0.05*	-
Time to targeted temperature	9,10,12,20	883	0.47 [0.02, 0.93]	0.04	86	-	-	-	-	-

^∗^Lactate clearance at all time-points did not differ among NMB groups (No specific data available).

ICU, intensive care unit; MV, mechanical ventilation.

## Discussion

This meta-analysis evaluated the safety and effectiveness of NMBA for CA survivors treated with TTM. The quality of the included studies was low to medium. The aggregated data showed a significant improvement in survival and neurological prognosis in prophylactic NMBA strategy compared to the absence of NMBA strategy. Subgroup analyses and sensitivity analyses confirmed these results. Also, there is no significant difference between the continuous NMBA and the bolus NMBA strategy. In addition, the NMBA strategy did not increase the patient’s hospital stay, duration of MV, the incidence of muscle weakness, and nosocomial infections.

### Comparison With Previous Research

Our study found that NMBA is widely used in clinical practice, but there are differences in the strategies used and their associated clinical outcomes. The prophylactic NMBA strategy was mostly applied among the included studies, which is in line with a previous systematic review. That article included 68 IUCs in which NMBA were routinely used to prevent shivering in 54 ICUs while treat shivering in eight ICUs (Chamorro et al., 2010).

The 2010 AHA guidelines for cardiopulmonary resuscitation and Emergency Cardiovascular Care stated that the duration of NMBA use should be minimized, and the NMBA depth should be monitored (Peberdy et al., 2010). However, these conclusions were inferences from expert opinion and other studies but not supported by clear evidence. The statement is prompted by concerns that NMDA might mask epileptic activity and limit neurological assessment. Since then, neither the AHA nor the European Resuscitation Council recommended routine use of NMBA during TTM in their 2015 guidelines (Callaway et al., 2015). In the latest clinical practice guidelines for continuous NMBA in critically ill adult patients, the routine use of NMBA is not recommended for patients receiving TTM after CA (insufficient evidence) (Murray et al., 2016). Meanwhile, it is recommended that NMBA can be used to treat significant shivering during TTM, a weak recommendation based on a post-hoc analysis of only one prospective observational study (111 patients in total) (Salciccioli et al., 2013).

In our study, we added 11 newly published studies with a total sample size of 11,317 patients (Jurado and Gulbis, 2011; Snider et al., 2012; Salciccioli et al., 2013; Curtis et al., 2014; Lascarrou et al., 2014; Lee et al., 2017; Stöckl et al., 2017; Lee et al., 2018; May et al., 2018; Moskowitz et al., 2020; Hifumi et al., 2021; Takiguchi et al., 2021). Although high-quality RCTs are still lacking, our sample size allowed for better statistical power and different sensitivities and subgroup analyses. The results of subgroup analyses basing on various NMBA strategies also confirm our findings’ robustness. In addition, our results showed that using NMBA is safe, i.e., NMBA does not increase the length of stay, duration of MV, nosocomial infections, or muscle weakness in CA patients receiving TTM. Thus, our study partially fills a gap in the previous guidelines and provides additional evidence for clinical NMBA application.

### Interpreting Our Findings

We found the prophylactic NMBA strategy significantly improved mortality and neurological outcome in CA survivors undergoing TTM. Several explanations might contribute to our findings. First, NMBA can effectively control shivering, which interferes with achieving target temperatures by generating heat and increases metabolic activity, oxygen consumption, and cerebral metabolic stress (De Witte and Sessler, 2002; Oddo et al., 2010). Several included studies reported reductions in shivering episodes during NMBA therapy (Stöckl et al., 2017; Moskowitz et al., 2020). Moskowitz et al. found approximately 40% of patients in the usual care group develop shivering and required NMBA rescue administration, while no shivering episodes were observed in the NMBA group Moskowitz et al. (2020). In another RCT, patients were randomized to receive either a continuous NMBA or an on-demand rocuronium bromide (Stöckl et al., 2017). The authors found that 94% of patients in the on-demand NMBA group had detectable shivering episodes compared to 25% receiving continuous rocuronium (*p* < 0.01) (Stöckl et al., 2017). The authors noted that shivering occurred throughout the TTM period, rather than just at a specific stage during the TTM course. In addition, shivering may also be invisible, manifesting as ECG artifacts, EMG activity, or delayed achievement of the target temperature (Seder et al., 2011). Thus, the prophylactic NMBA strategy may control invisible shivering, which attenuates the neuroprotective effects of TTM. Meanwhile, we should note one important potential bias in on-demand NMBA strategy, that is, shivering is a natural thermoregulatory response of the body to lowering the core temperature, but require the relatively intact brain function (Nair and Lundbye, 2013; Hovdenes et al., 2016). Thus, patients with more severe brain injury who did not present shivering would not gain NMBA when administered “on-demand” but would have worse outcomes due to more severe brain injury, not due to lack of NMBA.

Second, our findings suggest the safety of NMBA regimens. The previous controversy over the use of NMBA was that NMBA might be associated with the risk of early-onset pneumonia and critical illness polyneuropathy (Price et al., 2012; Lascarrou et al., 2014). It also increases the duration of MV and hospital stay. However, our findings did not reveal these results. With the development of technologies such as MV weaning, percutaneous tracheotomy, and the management of ventilator-associated pneumonia and cardiopulmonary resuscitation, most ICUs have clear protocols for managing MV during TTM and the prevention and control of nosocomial pneumonia (Callaway et al., 2015). This reduces the finding of positive clinical outcomes of adverse events in the NMBA and control groups. At the same time, the included studies showed that NMBA did not increase muscle weakness during their stay in ICU (Stöckl et al., 2017; Lee et al., 2018). This favorable result can also be partly explained by the short duration of NMBA use in all these studies (approximately 24–37 h) (Jurado and Gulbis, 2011; Snider et al., 2012; Salciccioli et al., 2013; Curtis et al., 2014; Lee et al., 2017; Lee et al., 2018; Moskowitz et al., 2020; Hifumi et al., 2021; Takiguchi et al., 2021). Similarly, a recently published meta-analysis of short-term NMBA application for ARDS treatment failed to find a correlation between NMBA and acquired muscle weakness (Tarazan et al., 2020).

However, we did not find a significant improvement in lactate levels after a prophylactic NMBA strategy. Previous theories believed that improved tissue perfusion and reduced metabolic demand were possible mechanisms for decreasing lactate levels following NMBA treatment (Salciccioli et al., 2013). Some authors explain that the duration of NMBA administration in the study was inconsistent across subjects, while the serum lactate levels were obtained at regular intervals at the specified times (Lascarrou et al., 2014). On the other hand, some patients in the control group also received a temporary bolus of NMBA for shivering episodes, which reduced lactate accumulation (Lee et al., 2018; Moskowitz et al., 2020). This may have weakened the perfusion and metabolic improvement effect in the NMDA group. We also found no significant reduction in the induction time of TTM, which might be due to the advances in cooling techniques and CPR management. As shown in the most included studies, the induction time was approximately 0.5–3 h, which might reduce shivering and other adverse events during that period (Curtis et al., 2014; Lee et al., 2017; Stöckl et al., 2017; Hifumi et al., 2021). Moreover, the initial lactate levels for the enrolling patients were not so high (1.4–3.6 mmol/L), which could partially explain the lack of differences in lactate clearance between groups (Salciccioli et al., 2013; Lascarrou et al., 2014; Lee et al., 2018; Moskowitz et al., 2020).

### Research Limitations

Our study has several limitations. First, most of the included studies were retrospective, which greatly affected the causality of our study findings. Second, some included studies also recruited patients with IHCA (Lascarrou et al., 2014; Lee et al., 2017; May et al., 2018; Moskowitz et al., 2020; Takiguchi et al., 2021), who might not benefit from TTM and even had a worse prognosis (Chan et al., 2016). Therefore, the value of NMBA for patients with IHCA still needs to be further explored. Third, there was considerable heterogeneity in the TTM regimens among the included studies in terms of cooling modalities, sedation drugs, timing, and methods of shivering monitoring. For example, apart from NMBA, other strategies to prevent or control shivering involve sedative or opioid administration, often used instead if NMBA is avoided or eliminated (May et al., 2018). Deep sedation can delay extubation, ICU transfer, lead to an increased incidence of delirium or infection, confound neurological assessment, perhaps even inappropriate withdrawal of life support (Samaniego et al., 2011; Barr et al., 2013; Sandroni et al., 2014). However, all the included studies had not provided the potential impact of assessing sedation or opioid changes during NMBA used in TTM. Fourth, although we used subgroup and sensitivity analyses to explore possible confounding factors, our results may have been influenced by unmeasured confounding factors; and the sample sizes for some of the subgroup analyses were small. Meanwhile, a sensitivity analysis that included only three small RCTs did not benefit from a preventive NMB strategy over a without NMBA strategy. Fifth, the included studies spanned an extensive range of periods, during which CPR and CA guidelines have been updated several times. Sixth, some secondary outcomes need to be treated with caution. For example, most retrospective studies may not have recognized mild or moderate weakness during routine clinical care. Thus, more studies focusing on this are required in the future. Finally, the included CA patients had different underlying diseases, demographic characteristics and used different disease severity scoring criteria. However, due to the number of studies, we could not perform subgroup analyses to clarify this point further.

## Conclusion

This meta-analysis indicates that prophylactic NMBA administration effectively reduces mortality and poor neurological outcome for comatose CA survivors during TTM. Continuous and intermittent NMBA has equal effectiveness in control shivering occurrence. However, due to the poor overall quality of current studies, further research with adequately powered RCTs is required to confirm our results.

## Data Availability

The original contributions presented in the study are included in the article/Supplementary Material, further inquiries can be directed to the corresponding author.

## Abbreviations

CA, cardiac arrest; CI, confidence interval; ICU, intensive care unit; IQR, interquartile range; LOS, length of stay; NMBA, neuromuscular-blocking agent; MD, mean difference; OHCA, out-of-hospital cardiac arrest; RR, risk ratio; RCTs, randomized controlled trials; SD, standard deviations; TTM, targeted temperature management.

## References

[B1] Al-Dorzi H. M. El-Saed A. Rishu A. H. Balkhy H. H. Memish Z. A. Arabi Y. M. (2012). The Results of a 6-year Epidemiologic Surveillance for Ventilator-Associated Pneumonia at a Tertiary Care Intensive Care Unit in Saudi Arabia. Am. J. Infect. Control 40 (9), 794–799. 10.1016/j.ajic.2011.10.004 22317860

[B2] Barr J. Fraser G. L. Puntillo K. Ely E. W. Gélinas C. Dasta J. F. (2013). Clinical Practice Guidelines for the Management of Pain, Agitation, and Delirium in Adult Patients in the Intensive Care Unit. Crit. Care Med. 41 (1), 263–306. 10.1097/CCM.0b013e3182783b72 23269131

[B3] Callaway C. W. Donnino M. W. Fink E. L. Geocadin R. G. Golan E. Kern K. B. (2015). Part 8: Post-Cardiac Arrest Care: 2015 American Heart Association Guidelines Update for Cardiopulmonary Resuscitation and Emergency Cardiovascular Care. Circulation 132 (18 Suppl. 2), S465–S482. 10.1161/CIR.0000000000000262 26472996 PMC4959439

[B4] Chamorro C. Borrallo J. M. Romera M. A. Silva J. A. Balandín B. (2010). Anesthesia and Analgesia Protocol during Therapeutic Hypothermia after Cardiac Arrest: a Systematic Review. Anesth. Analg. 110 (5), 1328–1335. 10.1213/ANE.0b013e3181d8cacf 20418296

[B5] Chan P. S. Berg R. A. Tang Y. Curtis L. H. Spertus J. A. (2016). Association between Therapeutic Hypothermia and Survival after In-Hospital Cardiac Arrest. Jama 316 (13), 1375–1382. 10.1001/jama.2016.14380 27701659 PMC5486217

[B6] Curtis J. Zettlemoyer G. Butler I. (2014). Neuromuscular Blocking Agents Do Not Impact Time to Target Temperature with Therapeutic Hypothermia. Crit. Care Med. 42 (12 Suppl. L), 1. (A1421-). 10.1097/01.ccm.0000457749.54484.61 24105456

[B7] De Witte J. Sessler D. I. (2002). Perioperative Shivering: Physiology and Pharmacology. Anesthesiology 96 (2), 467–484. 10.1097/00000542-200202000-00036 11818783

[B8] deBacker J. Hart N. Fan E. (2017). Neuromuscular Blockade in the 21st Century Management of the Critically Ill Patient. Chest 151 (3), 697–706. 10.1016/j.chest.2016.10.040 27818334

[B9] Greenberg S. B. Vender J. (2013). The Use of Neuromuscular Blocking Agents in the ICU: where Are We Now? Crit. Care Med. 41 (5), 1332–1344. 10.1097/CCM.0b013e31828ce07c 23591211

[B10] Hifumi T. Inoue A. Arimoto H. Yonemoto N. Kuroda Y. Tahara Y. (2021). The Association between Neuromuscular Blockade Use during Target Temperature Management and Neurological Outcomes. Am. J. Emerg. Med. 46, 289–294. 10.1016/j.ajem.2020.07.078 33051089

[B11] Higgins J. P. Altman D. G. Gøtzsche P. C. Jüni P. Moher D. Oxman A. D. (2011). The Cochrane Collaboration's Tool for Assessing Risk of Bias in Randomised Trials. BMJ 343, d5928. 10.1136/bmj.d5928 22008217 PMC3196245

[B12] Higgins J. P. Thompson S. G. Deeks J. J. Altman D. G. (2003). Measuring Inconsistency in Meta-Analyses. BMJ 327 (7414), 557–560. 10.1136/bmj.327.7414.557 12958120 PMC192859

[B13] Hovdenes J. Røysland K. Nielsen N. Kjaergaard J. Wanscher M. Hassager C. (2016). A Low Body Temperature on Arrival at Hospital Following Out-Of-Hospital-Cardiac-Arrest Is Associated with Increased Mortality in the TTM-Study. Resuscitation 107, 102–106. 10.1016/j.resuscitation.2016.08.011 27565034

[B14] Jurado L. V. Gulbis B. E. (2011). Continuous Infusion versus Intermittent Bolus Dosing of Vecuronium in Patients Receiving Therapeutic Hypothermia after Sudden Cardiac Arrest. Pharmacotherapy 31 (12), 1250–1256. 10.1592/phco.31.12.1250 22122185

[B15] Lascarrou J. B. Le Gouge A. Dimet J. Lacherade J. C. Martin-Lefèvre L. Fiancette M. (2014). Neuromuscular Blockade during Therapeutic Hypothermia after Cardiac Arrest: Observational Study of Neurological and Infectious Outcomes. Resuscitation 85 (9), 1257–1262. 10.1016/j.resuscitation.2014.05.017 24892266

[B16] Lee B. K. Cho I. S. Oh J. S. Choi W. J. Wee J. H. Kim C. S. (2018). Continuous Neuromuscular Blockade Infusion for Out-Of-Hospital Cardiac Arrest Patients Treated with Targeted Temperature Management: A Multicenter Randomized Controlled Trial. PloS one 13 (12), e0209327. 10.1371/journal.pone.0209327 30557377 PMC6296517

[B17] Lee D. H. Lee B. K. Jeung K. W. Jung Y. H. Cho Y. S. Youn C. S. (2017). Neuromuscular Blockade Requirement Is Associated with Good Neurologic Outcome in Cardiac Arrest Survivors Treated with Targeted Temperature Management. J. Crit. Care 40, 218–224. 10.1016/j.jcrc.2017.04.026 28448951

[B18] May T. L. Riker R. R. Fraser G. L. Hirsch K. G. Agarwal S. Duarte C. (2018). Variation in Sedation and Neuromuscular Blockade Regimens on Outcome after Cardiac Arrest. Crit. Care Med. 46 (10), e975–e980. 10.1097/CCM.0000000000003301 29979225 PMC6138551

[B19] Moskowitz A. Andersen L. W. Rittenberger J. C. Swor R. Seethala R. R. Kurz M. C. (2020). Continuous Neuromuscular Blockade Following Successful Resuscitation from Cardiac Arrest: A Randomized Trial. J. Am. Heart Assoc. 9 (17), e017171. 10.1161/JAHA.120.017171 32851921 PMC7660770

[B20] Murray M. J. DeBlock H. Erstad B. Gray A. Jacobi J. Jordan C. (2016). Clinical Practice Guidelines for Sustained Neuromuscular Blockade in the Adult Critically Ill Patient. Crit. Care Med. 44 (11), 2079–2103. 10.1097/CCM.0000000000002027 27755068

[B21] Nair S. U. Lundbye J. B. (2013). The Occurrence of Shivering in Cardiac Arrest Survivors Undergoing Therapeutic Hypothermia Is Associated with a Good Neurologic Outcome. Resuscitation 84 (5), 626–629. 10.1016/j.resuscitation.2012.11.018 23201502

[B22] Oddo M. Frangos S. Maloney-Wilensky E. Andrew Kofke W. Le Roux P. D. Levine J. M. (2010). Effect of Shivering on Brain Tissue Oxygenation during Induced Normothermia in Patients with Severe Brain Injury. Neurocrit Care 12 (1), 10–16. 10.1007/s12028-009-9280-2 19821062

[B23] Peberdy M. A. Callaway C. W. Neumar R. W. Geocadin R. G. Zimmerman J. L. Donnino M. (2010). Part 9: Post-cardiac Arrest Care: 2010 American Heart Association Guidelines for Cardiopulmonary Resuscitation and Emergency Cardiovascular Care. Circulation 122 (18 Suppl. 3), S768–S786. 10.1161/CIRCULATIONAHA.110.971002 20956225

[B24] Price D. Kenyon N. J. Stollenwerk N. (2012). A Fresh Look at Paralytics in the Critically Ill: Real Promise and Real Concern. Ann. Intensive Care 2 (1), 43. 10.1186/2110-5820-2-43 23062076 PMC3519794

[B25] Salciccioli J. Donnino M. (2014). Reply to Letter: Continuous Neuromuscular Blockade Is Associated with Decreased Mortality in Post-cardiac Arrest Patients-Pproblems with the Data. Resuscitation 85 (1), e3. 10.1016/j.resuscitation.2013.09.011 24056393

[B26] Salciccioli J. D. Cocchi M. N. Rittenberger J. C. Peberdy M. A. Ornato J. P. Abella B. S. (2013). Continuous Neuromuscular Blockade Is Associated with Decreased Mortality in Post-cardiac Arrest Patients. Resuscitation 84 (12), 1728–1733. 10.1016/j.resuscitation.2013.06.008 23796602 PMC3864774

[B27] Samaniego E. A. Mlynash M. Caulfield A. F. Eyngorn I. Wijman C. A. (2011). Sedation Confounds Outcome Prediction in Cardiac Arrest Survivors Treated with Hypothermia. Neurocrit Care 15 (1), 113–119. 10.1007/s12028-010-9412-8 20680517 PMC3153345

[B28] Sandroni C. Cariou A. Cavallaro F. Cronberg T. Friberg H. Hoedemaekers C. (2014). Prognostication in Comatose Survivors of Cardiac Arrest: an Advisory Statement from the European Resuscitation Council and the European Society of Intensive Care Medicine. Resuscitation 85 (12), 1779–1789. 10.1016/j.resuscitation.2014.08.011 25438253

[B29] Seder D. B. May T. Fraser G. L. Riker R. R. (2011). Shivering during Therapeutic Hypothermia after Cardiac Arrest. Resuscitation 82 (2), 149. 10.1016/j.resuscitation.2010.09.012 21093972

[B30] Shamseer L. Moher D. Clarke M. Ghersi D. Liberati A. Petticrew M. (2015). Preferred Reporting Items for Systematic Review and Meta-Analysis Protocols (PRISMA-P) 2015: Elaboration and Explanation. BMJ 350, g7647. 10.1136/bmj.g7647 25555855

[B31] Snider J. Vandenberg M. Thomas W. Bockheim H. (2012). 573. Crit. Care Med. 40 1–328. 10.1097/01.ccm.0000424791.82973.a9 23213646

[B32] Stang A. (2010). Critical Evaluation of the Newcastle-Ottawa Scale for the Assessment of the Quality of Nonrandomized Studies in Meta-Analyses. Eur. J. Epidemiol. 25 (9), 603–605. 10.1007/s10654-010-9491-z 20652370

[B33] Stöckl M. Testori C. Sterz F. Holzer M. Weiser C. Schober A. (2017). Continuous versus Intermittent Neuromuscular Blockade in Patients during Targeted Temperature Management after Resuscitation from Cardiac Arrest-A Randomized, Double Blinded, Double Dummy, Clinical Trial. Resuscitation 120, 14–19. 10.1016/j.resuscitation.2017.08.238 28860012

[B34] Takiguchi T. Ohbe H. Nakajima M. Sasabuchi Y. Tagami T. Matsui H. (2021). Intermittent versus Continuous Neuromuscular Blockade during Target Temperature Management after Cardiac Arrest: A Nationwide Observational Study. J. Crit. Care 62, 276–282. 10.1016/j.jcrc.2021.01.002 33508762

[B35] Tarazan N. Alshehri M. Sharif S. Al Duhailib Z. Møller M. H. Belley-Cote E. (2020). Neuromuscular Blocking Agents in Acute Respiratory Distress Syndrome: Updated Systematic Review and Meta-Analysis of Randomized Trials. Intensive Care Med. Exp. 8 (1), 61. 10.1186/s40635-020-00348-6 33095344 PMC7582438

[B36] Wan X. Wang W. Liu J. Tong T. (2014). Estimating the Sample Mean and Standard Deviation from the Sample Size, Median, Range And/or Interquartile Range. BMC Med. Res. Methodol. 14, 135. 10.1186/1471-2288-14-135 25524443 PMC4383202

