# Supplementary material for: Neuromuscular Blockade for Cardiac Arrest Patients Treated With Targeted Temperature Management: A Systematic Review and Meta-Analysis
Source: Front Pharmacol. 2022 May 24;13:780370. doi: 10.3389/fphar.2022.780370 (PMC9171045; doi:10.3389/fphar.2022.780370)
Supplement: Supplementary file 1 [file DataSheet1.docx]

**Neuromuscular blockade for cardiac arrest patients treated with targeted temperature management: A systematic review and meta-analysis**

Tong Lin; MD; Yan Yao, MD; Yuan Xu, MD; Hui-Bin Huang*, MD

Corresponding author: Hui-Bin Huang, Email: hhba02922@btch.edu.cn

**Additional files**

Additional file 1 PRISMA checklist……………………………………………………………………………………………………………………………………………………………………………………………….……………………………………..2

Additional file 2 Search Strategy………………………………………………………………………………………………………………………………………………………………………………………………………………….……………………..5

Additional file 3 Definition of inclusion and exclusion criteria for patient population and regimens of conservative and conventional oxygen………………………………………..………………...……..8

Additional file 4 Description of sedative/anesthetic strategies and treatment among the included studies……………………………………………………………………………………………………………………..10

Additional file 5 Cochrane risk of bias………………………………………………………………………………………………………………………………………………………………………………………………………………….…....……..11

Additional file 6 Subgroup analysis…...………………………..……………………………..……………………….………………………………………………………………………………..………………………………………….…………....…13

**Additional file 1**

**PRISMA 2009 checklist**

| **Section/topic** | **#** | **Checklist item** | **Reported on page #** |
| --- | --- | --- | --- |
| **TITLE** | | |  |
| Title | 1 | Identify the report as a systematic review, meta-analysis, or both. | 1 |
| **ABSTRACT** | | |  |
| Structured summary | 2 | Provide a structured summary including, as applicable: background; objectives; data sources; study eligibility criteria, participants, and interventions; study appraisal and synthesis methods; results; limitations; conclusions and implications of key findings; systematic review registration number. | 2 |
| **INTRODUCTION** | | |  |
| Rationale | 3 | Describe the rationale for the review in the context of what is already known. | 4 |
| Objectives | 4 | Provide an explicit statement of questions being addressed with reference to participants, interventions, comparisons, outcomes, and study design (PICOS). | 4-5 |
| **METHODS** | | |  |
| Protocol and registration | 5 | Indicate if a review protocol exists, if and where it can be accessed (e.g., Web address), and, if available, provide registration information including registration number. | 6 |
| Eligibility criteria | 6 | Specify study characteristics (e.g., PICOS, length of follow-up) and report characteristics (e.g., years considered, language, publication status) used as criteria for eligibility, giving rationale. | 6 |
| Information sources | 7 | Describe all information sources (e.g., databases with dates of coverage, contact with study authors to identify additional studies) in the search and date last searched. | 6 |
| Search | 8 | Present full electronic search strategy for at least one database, including any limits used, such that it could be repeated. | 6 and Aditional 2 |
| Study selection | 9 | State the process for selecting studies (i.e., screening, eligibility, included in systematic review, and, if applicable, included in the meta-analysis). | 6 |
| Data collection process | 10 | Describe method of data extraction from reports (e.g., piloted forms, independently, in duplicate) and any processes for obtaining and confirming data from investigators. | 6-7 |
| Data items | 11 | List and define all variables for which data were sought (e.g., PICOS, funding sources) and any assumptions and simplifications made. | 7 |
| Risk of bias in individual studies | 12 | Describe methods used for assessing risk of bias of individual studies (including specification of whether this was done at the study or outcome level), and how this information is to be used in any data synthesis. | 7 |
| Summary measures | 13 | State the principal summary measures (e.g., risk ratio, difference in means). | 7 |
| Synthesis of results | 14 | Describe the methods of handling data and combining results of studies, if done, including measures of consistency (e.g., I^2^) for each meta-analysis. | 7-8 |

| Risk of bias across studies | 15 | Specify any assessment of risk of bias that may affect the cumulative evidence (e.g., publication bias, selective reporting within studies). | 8 |
| --- | --- | --- | --- |
| Additional analyses | 16 | Describe methods of additional analyses (e.g., sensitivity or subgroup analyses, meta-regression), if done, indicating which were pre-specified. | 8 |
| **RESULTS** | | |  |
| Study selection | 17 | Give numbers of studies screened, assessed for eligibility, and included in the review, with reasons for exclusions at each stage, ideally with a flow diagram. | 9 |
| Study characteristics | 18 | For each study, present characteristics for which data were extracted (e.g., study size, PICOS, follow-up period) and provide the citations. | 9, Table 1,  Additional 3 |
| Risk of bias within studies | 19 | Present data on risk of bias of each study and, if available, any outcome level assessment (see item 12). | 9  Additional 4 |
| Results of individual studies | 20 | For all outcomes considered (benefits or harms), present, for each study: (a) simple summary data for each intervention group (b) effect estimates and confidence intervals, ideally with a forest plot. | 9 |
| Synthesis of results | 21 | Present results of each meta-analysis done, including confidence intervals and measures of consistency. | 9-11 |
| Risk of bias across studies | 22 | Present results of any assessment of risk of bias across studies (see Item 15). | Additional 4 |
| Additional analysis | 23 | Give results of additional analyses, if done (e.g., sensitivity or subgroup analyses, meta-regression [see Item 16]). | 10-11  Table 2-3 |
| **DISCUSSION** | | |  |
| Summary of evidence | 24 | Summarize the main findings including the strength of evidence for each main outcome; consider their relevance to key groups (e.g., healthcare providers, users, and policy makers). | 12-15 |
| Limitations | 25 | Discuss limitations at study and outcome level (e.g., risk of bias), and at review-level (e.g., incomplete retrieval of identified research, reporting bias). | 15 |
| Conclusions | 26 | Provide a general interpretation of the results in the context of other evidence, and implications for future research. | 17 |
| **FUNDING** | | |  |
| Funding | 27 | Describe sources of funding for the systematic review and other support (e.g., supply of data); role of funders for the systematic review. | 18 |

**Additional file 2**

**Search Strategy：**

**Database: PubMed, Embase, Cochrane library；**

**Search completed on 15th Jul 2021.**

=====================================================================================================================

**PubMed** 58

("Neuromuscular Blockade"[MeSH Terms] OR "Neuromuscular Blocking Agents"[MeSH Terms] OR ("Neuromuscular Blockade"[Title/Abstract] OR "Neuromuscular Blocking Agents"[Title/Abstract] OR ("vecuronium"[Title/Abstract] OR "muscle relaxant"[Title/Abstract] OR "rocuronium"[Title/Abstract] OR "atracurium"[Title/Abstract] OR "cis-atracurium"[Title/Abstract] OR "vecuronium"[Title/Abstract] OR "mivacurium"[Title/Abstract] OR "suxamethonium"[Title/Abstract] OR "succinylcholine"[Title/Abstract] OR "rapacuronium"[Title/Abstract] OR "pancuronium"[Title/Abstract] OR "skeletal muscle relaxant"[Title/Abstract]))) AND (("target temperature management"[Title/Abstract] OR "therapeutic hypothermia"[Title/Abstract] OR "hypothermia, induced"[MeSH Terms]) AND ("death, sudden, cardiac"[MeSH Terms] OR "Out-of-Hospital Cardiac Arrest"[MeSH Terms] OR "Heart Arrest"[MeSH Terms] OR "cardiac arrest"[Title/Abstract]))

**-------------------------------------------------------------------------------------------------------------------------------------------------------------------------------------------------------**

**Embase** -123

Embase

No. Query Results

#25. #16 AND #20 AND #24

#24. #21 OR #22 OR #23

#23. 'heart arrest':ab,ti

#22. 'cardiac arrest':ab,ti

#21. 'heart arrest'/exp

#20. #17 OR #18 OR #19

#19. 'induced hypothermia'/exp

#18. 'therapeutic hypothermia':ab,ti

#17. 'target temperature management':ab,ti

#16. #1 OR #2 OR #3 OR #4 OR #5 OR #6 OR #7 OR #8 OR #9 OR #10 OR #11 OR #12 OR #13 OR #14 OR #15

#15. 'skeletal muscle relaxant':ab,ti

#14. 'pancuronium':ab,ti

#13. 'rapacuronium':ab,ti

#12. 'succinylcholine':ab,ti

#11. 'suxamethonium':ab,ti

#10. 'mivacurium':ab,ti

#9. 'vecuronium':ab,ti

#8. 'cis-atracurium':ab,ti

#7. 'atracurium':ab,ti

#6. 'rocuronium':ab,ti

#5. 'muscle relaxant':ab,ti

#4. 'vecuronium':ab,ti

#3. 'neuromuscular blocking agents':ab,ti

#2. 'neuromuscular blockade':ab,ti

#1. 'neuromuscular blocking'/exp

---------------------------------------------------------------------------------------------------------------------------------------------------------------------------------------------------------

**Cochrane library** -15

ID Search

#1 Neuromuscular Blockade

#2 MeSH descriptor: [Neuromuscular Blockade] explode all trees

#3 Neuromuscular Blocking Agents

#4 MeSH descriptor: [Neuromuscular Blocking Agents] explode all trees

#5 vecuronium

#6 muscle relaxant

#7 rocuronium

#8 atracurium

#9 cis-atracurium

#10 vecuronium

#11 mivacurium

#12 suxamethonium

#13 succinylcholine

#14 rapacuronium

#15 pancuronium

#16 skeletal muscle relaxant

#17 #1 OR #2 OR #3 OR #4 OR #5 OR #6 OR #7 OR #8 OR #9 OR #10 OR #11 OR #12 OR #13 OR #14 OR #15 OR #16

#18 target temperature management

#19 MeSH descriptor: [Hypothermia, Induced] explode all trees

#20 therapeutic hypothermia

#21 #18 OR #19 OR #20

#22 Cardiac Arrest

#23 MeSH descriptor: [Heart Arrest] explode all trees

#24 heart arrest

#25 death, sudden, cardiac

#26 MeSH descriptor: [Death, Sudden, Cardiac] explode all trees

#27 #22 OR #23 OR #24 OR #25 OR #26

#28 #17 AND #21 AND #

**Additional file 3**

**Table S3：Definition of inclusion and exclusion criteria for patient population and regimens of NMBA**

| Study / Year | Prophylactic NMBA regimen | Bolus if demanded | Absence of NMBA regimen |
| --- | --- | --- | --- |
| Takiguchi 2021 | We defined the **continuous** NMBA group as patients who received either rocuronium (≥250 mg/day) or vecuronium (≥50 mg/day) on the day of admission. | We defined the intermittent NMBA group as those who received NMBAs **as required of** either rocuronium (>50 mg/day and < 250 mg/day) or vecuronium (>10 mg/day and < 50 mg/day) on the day of admission. |  |
| Stöckl 2017 | Patients randomized to the **continuous** NMB group received rocuronium with an initial bolus of 0.25mg/kg of bodyweight followed by a continuous application of 0.25 mg/kg/h. |  | Patients in the bolus-NMB-group received an initial bolus of **saline** as well as a subsequent continuous application of **saline**. |
| Lee 2018 | Subjects assigned to the NMB group received a bolus of 0.6 mg/kg rocuronium at the beginning of TTM, **followed by an infusion** of 0.3–0.6 mg/kg/hr. |  | Subjects assigned to the **placebo** group received isotonic **saline** without NMB for 24 hours after assignment. the placebo group was allowed to receive bolus NMB in cases of intractable shivering |
| May 2018 | Sedation with **continuous or scheduled** **NMB** | Sedation with **as-needed** NMB. | …**escalating** sedation dosing and **avoidance of** NMB |
| Lee 2017 | Based on NMB requirements, subjects were divided into three groups: no NMB, bolus NMB, and **continuous NMB**. | Based on NMB requirements, subjects were divided into three groups: no NMB, **bolus NMB**, and continuous NMB. | Based on NMB requirements, subjects were divided into three groups: **no NMB,** bolus NMB, and continuous NMB. |
| Hifumi 2020 | Therefore, the dose, administration method (**intermittent or continuous**), and types of NMB varied among the participating hospitals |  | **No NMB** |
| Moskowitz 2020 | Patients in the **continuous NMB** arm received a rocuronium bolus of 1.0 mg/kg followed by a continuous infusion of rocuronium for a total of 24 hours titrated to 1 to 2/4 twitches on a train-of-4 stimulator. |  | Patients in the usual care arm received 100 mL of **normal (0.9% NaCl) saline** to mark the 0-hour time point. |
| Lascarrou 2014 | intravenous bolus of 10 mg of the cisatracurium; **continuous** cisatracurium infusion in an initial dose of 10 mg/h. |  | **No NMB** therapy. |
| Snider 2012 | The objective of this study is to compare time to goal temperature among three groups: **continuous infusion NMBA** (65/98, 66%), **intermittent NMBA** (21/98, 21%), and no NMBA to prevent shivering during therapeutic hypothermia post cardiac arrest. |  | The objective of this study is to compare time to goal temperature among three groups: continuous infusion NMBA, intermittent NMBA and **no NMBA** (12/98, 12%) to prevent shivering during therapeutic hypothermia post cardiac arrest. |
| Curtis J 2014 | Patients were divided into two groups based on the **presence of any NMBA** use during the first 24 hours of TH. |  | Patients were divided into two groups based on the **absence of** any NMBA use during the first 24 hours of TH. |
| Jurado 2011 | The **vecuronium continuous-infusion** group and the  vecuronium bolus group. The continuous infusion  group consisted of patients treated with a vecuronium continuous infusion at 0.8 μg/kg/minute titrated to a goal TOF response of 1/4–2/4 twitches every hour until a consistent level was reached. | The bolus group consisted of those patients treated with vecuronium intermittent boluses at a dose of 0.05 mg/kg every 1 hour **as needed** for any shivering in the emergency department and cardiac catheterization laboratory and then 0.05 mg/kg every 2 hours as needed to achieve a goal TOF response of 1/4–2/4 twitches or for visible movement in the cardiac care unit. |  |
| Salciccioli 2013 | A portion of these patients (18/111, 16%) patients had NMB initiated immediately and **sustained for a minimum** duration of 24 hours following return of circulation. | In patients (93/111, 84%) **without sustained** NMB… |  |

**Additional file 4**

**Table S4：Description of sedative/anesthetic strategies and treatment among the included studies**

| Study / Year | Sedative/anesthetic strategies | Sedative/anesthetic treatment |
| --- | --- | --- |
| Takiguchi 2021^[18]^ | NA | NA |
| Stöckl 2017^[9]^ | Sedation was induced by the intravenous administration of **midazolam** (0.125mg/kg/h) and **fentanyl** (2 μg/kg/h). An additional bolus of sedative was allowed if clinically indicated. If shivering (SAS ≥ 1) was detected, a blinded bolus medication was administered, and sedation was increased as an equivalent of an additional bodyweight of 5 kg (accordingly by an increase of midazolam of 0.625mg/h and fentanyl of 10 μg/h). The study medication was stopped at 29 hours and the sedation and analgesia at 31 hours after initiation of cooling (Figure 1). | Patients randomized to the continuous-NMB-group received significant lower doses of **midazolam** (4.3±0.8mg/kg vs. 5.1±0.9mg/kg, p<0.01) and **fentanyl** (0.062±0.014mg/kg vs. 0.071±0.007mg/kg, p<0.01), but required higher cumulative doses of rocuronium (7.8±1.8mg/kg vs. 2.3±1.6mg/kg, p<0.01). |
| Lee 2018^[11]^ | According to the standard guidelines | The requirements of vasopressors, **sedatives, and analgesics** were not different between the NMB and placebo groups. |
| May 2018^[19]^ | SP1 indicated escalating **sedation** dosing and avoidance of NMB, SP2 indicated sedation with either scheduled or continuous NMB to prevent shivering, and SP3 indicated sedation with as-needed NMB in response to shivering | NA |
| Lee 2017^[20]^ | Remifentanil and midazolam were routinely used for sedation and analgesia. | NA |
| Hifumi 2020^[12]^ | NA | More NMB group patients received **sedative/anesthetic** treatment than no NMB group patients |
| Moskowitz 2020^[10]^ | All patients, including those who received continuous NMB, were sedated per local site protocol. | Propofol, midazolam, lorazepam, dexmedetomidine, and fentanyl were used and were comparable between the NMB and usual care groups. |
| Lascarrou 2014^[5]^ | All patients were sedated with **midazolam**. Doses were adjusted to obtain a RASS score of −5. When shivering occurs, a single intravenous bolus of a hypnotic agent and an opioid in a dose that depends on the infusion rates of hypnotic and opioid drugs (i.e., midazolam 5-mg intravenous bolus if the continuous midazolam infusion rate was 5 mg/h). | NA |
| Snider 2012 | NA | NA |
| Curtis J 2014^[22]^ | NA | NA |
| Jurado 2011^[21]^ | NA | NA |
| Salciccioli 2013^[18]^ | NA | NA |

**Additional file 5**

**Table S5: Quality assessment and overall risk of bias of included studies (for observational studies)**

| First author / year | Patient selection | | | | Comparability | Outcome | | | Risk of bias |
| --- | --- | --- | --- | --- | --- | --- | --- | --- | --- |
|  | Representation of the exposed cohort | Selection of the non-exposed cohort | Ascertainment of exposure | Outcome of  interest not  present at start | Comparability of cohorts on the basis of the design or analysis | Assessment  of outcome | Was follow-up long enough for outcomes to occur | Adequacy of follow up of cohorts |  |
| Hifumi 2020^[12]^ | ★ | ★ | ★ | ☆ | ★★ | ★ | ★ | ★ | 8 |
| Takiguchi 2021^[8]^ | ★ | ★ | ★ | ☆ | ★★ | ★ | ★ | ★ | 8 |
| May 2018^[19]^ | ★ | ★ | ★ | ☆ | ☆★ | ★ | ★ | ★ | 7 |
| Lee 2017^[20]^ | ★ | ★ | ★ | ☆ | ★★ | ★ | ★ | ★ | 8 |
| Lascarrou 2014^[5]^ | ★ | ★ | ★ | ☆ | ☆★ | ★ | ★ | ★ | 7 |
| Curtis 2014^[22]^ | ★ | ★ | ★ | ☆ | ☆☆ | ★ | ★ | ★ | 6 |
| Snider 2012^[23]^ | ★ | ★ | ★ | ☆ | ☆★ | ★ | ★ | ★ | 7 |
| Salciccioli 2013^[18]^ | ★ | ★ | ★ | ☆ | ☆★ | ★ | ★ | ★ | 7 |
| Jurado 2011^[21]^ | ★ | ★ | ★ | ☆ | ☆☆ | ★ | ★ | ★ | 6 |

**Abbreviations:** H=high quality; M=moderate quality; L= low quality.

**Note:** A study was given a maximum of one point in each item within the Patient selection and Outcome domains and given a maximum of two points for the Comparability domain with the following criteria:

1. **Representation of the exposed cohort**：Studies received 1 point if they recruited consecutive series of adult patients with the TTM, or all included patients or did not miss a large number of patients.

2. **Selection of the non-exposed cohort**：Studies received 1 point if both groups of patients with or without the TTM were recruited from the same cohort.

3. **Ascertainment of exposure**: Studies received 1 point if they use the TTM technique, regardless of cooling method, duration of TTM, induced time or others, to treat patients with cardiac arrests.

4. **Outcome of interest was not present at start of study**: Studies received points if they demonstrated the outcome of interest was not present at the start of the study.

5. **Comparability:** Studies received points if they controlled the initial rhythm, that is, shockable or non-shockable rhythm, (1 point); or any additional important factors such as basal CPC levels before TTM, IHCA or OHCA, targeted temperature, or there were no significant differences between patients with or without TTM treatment (1 point).

6. **Assessment of outcome**: Studies received 1 point if they had independent blind assessment or record linkage.

7. **Was follow-up long enough for outcomes to occur**: Studies received 1 point if they follow up until at least either inpatient mortality or for 30 days or had adequate record linkage.

8. **Adequacy of follow up for cohorts**: Studies received 1 point if all recruited subjects were all followed up, or the number lost to follow-up was unlikely to introduce bias (≤10%).

===================================================================================================================

**Cochrane risk of bias (for RCTs)**

**- Risk of bias graph: review authors' judgements about each risk of bias item presented as percentages across all included studies.(Left figure)**

**- Risk of bias summary: review authors' judgements about each risk of bias item for each included study. (Right figure)**


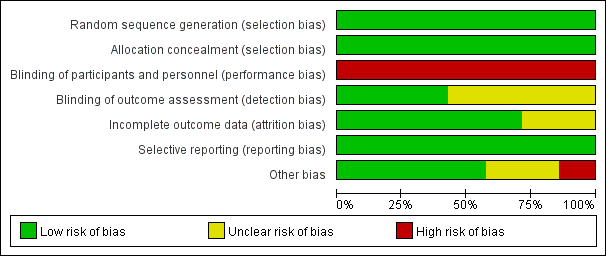

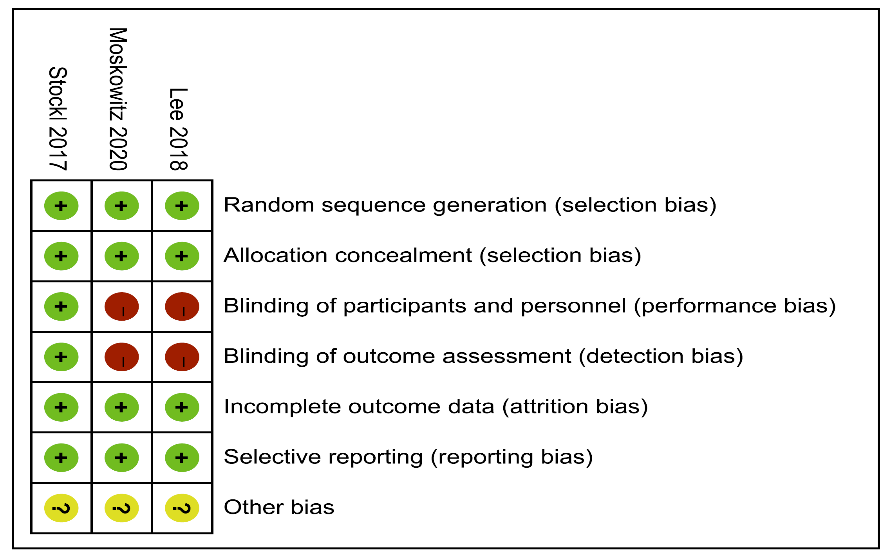


**Additional file 6**


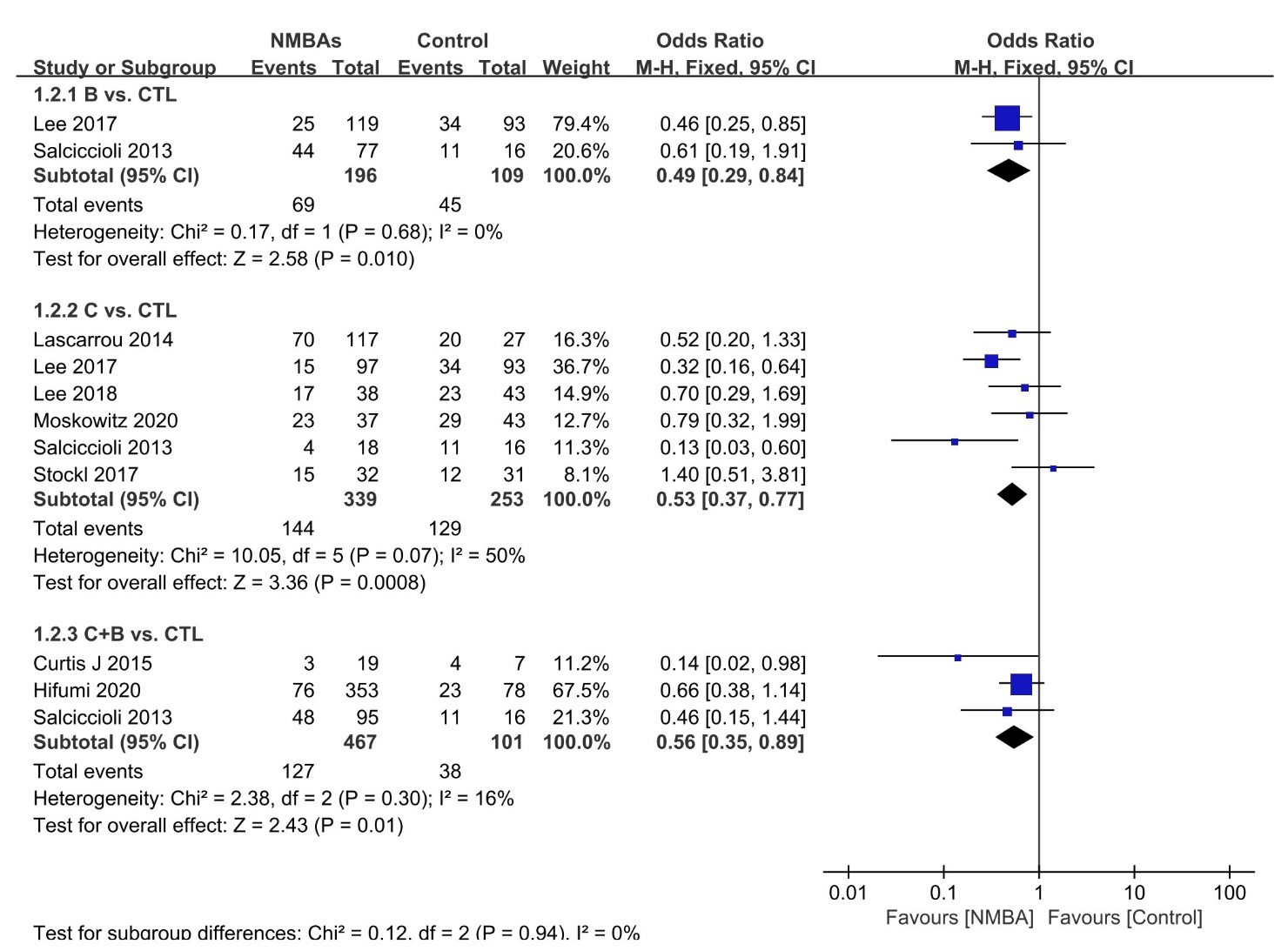


**Figure**: Subgroup analyses f outcome of mortality rate based on NMBA regimens.


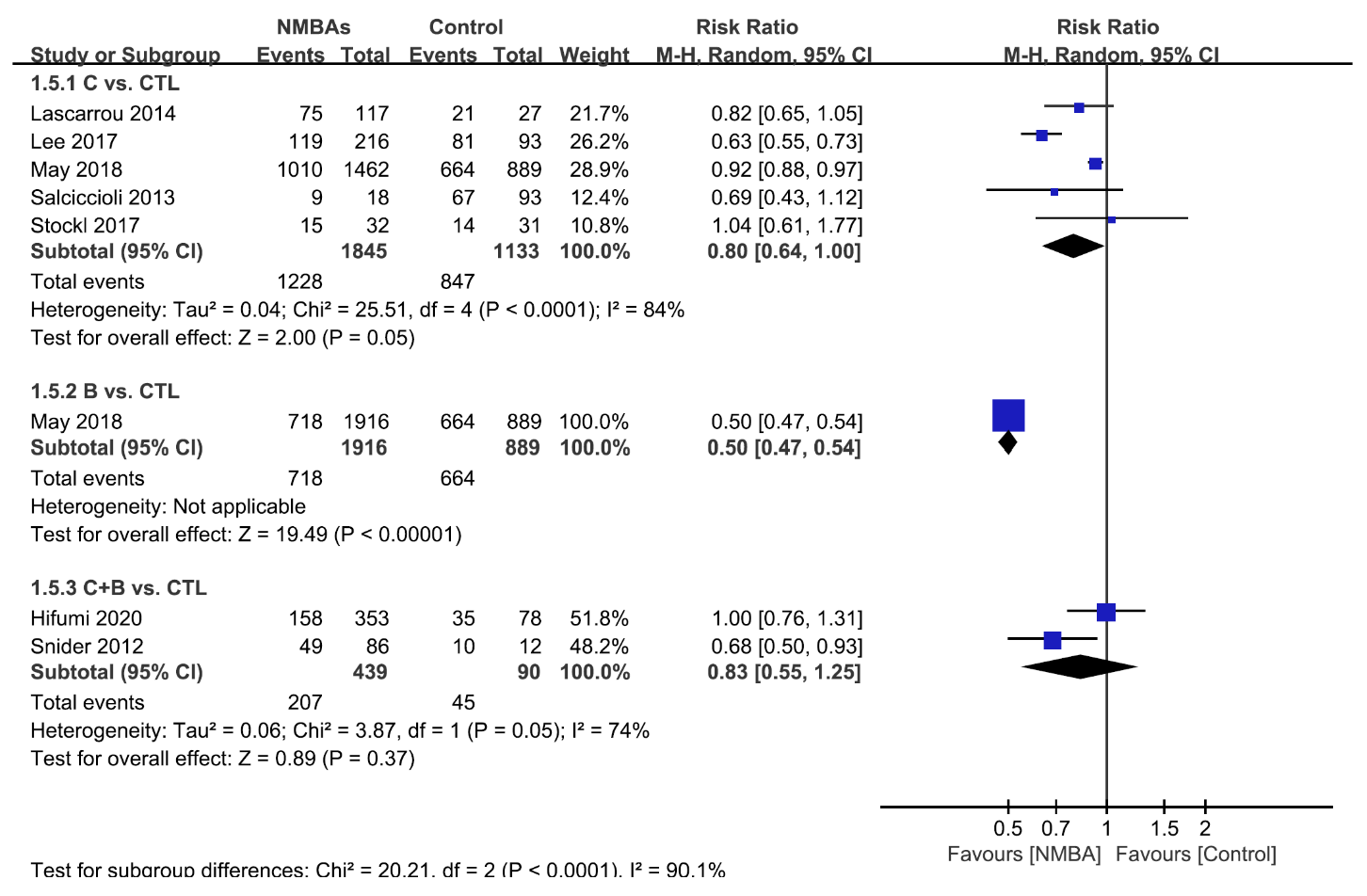


**Figure**: The subgroup analyses showed significant reductions in poor neurological outcomes.
